# Benchmarking short sequence mapping tools

**DOI:** 10.1186/1471-2105-14-184

**Published:** 2013-06-07

**Authors:** Ayat Hatem, Doruk Bozdağ, Amanda E Toland, Ümit V Çatalyürek

**Affiliations:** 1 Department of Electrical and Computer Engineering, The Ohio State University, Columbus, OH, USA; 2 Department of Biomedical Informatics, The Ohio State University, Columbus, OH, USA; 3 Department of Molecular Virology, Immunology and Medical Genetics, The Ohio State University, Columbus, OH, USA

**Keywords:** Short sequence mapping, Next-generation sequencing, Benchmark, Sequence analysis

## Abstract

**Background:**

The development of next-generation sequencing instruments has led to the generation of millions of short sequences in a single run. The process of aligning these reads to a reference genome is time consuming and demands the development of fast and accurate alignment tools. However, the current proposed tools make different compromises between the accuracy and the speed of mapping. Moreover, many important aspects are overlooked while comparing the performance of a newly developed tool to the state of the art. Therefore, there is a need for an objective evaluation method that covers all the aspects. In this work, we introduce a benchmarking suite to extensively analyze sequencing tools with respect to various aspects and provide an objective comparison.

**Results:**

We applied our benchmarking tests on 9 well known mapping tools, namely, Bowtie, Bowtie2, BWA, SOAP2, MAQ, RMAP, GSNAP, Novoalign, and mrsFAST (mrFAST) using synthetic data and real RNA-Seq data. MAQ and RMAP are based on building hash tables for the reads, whereas the remaining tools are based on indexing the reference genome. The benchmarking tests reveal the strengths and weaknesses of each tool. The results show that no single tool outperforms all others in all metrics. However, Bowtie maintained the best throughput for most of the tests while BWA performed better for longer read lengths. The benchmarking tests are not restricted to the mentioned tools and can be further applied to others.

**Conclusion:**

The mapping process is still a hard problem that is affected by many factors. In this work, we provided a benchmarking suite that reveals and evaluates the different factors affecting the mapping process. Still, there is no tool that outperforms all of the others in all the tests. Therefore, the end user should clearly specify his needs in order to choose the tool that provides the best results.

## Introduction

Next-generation sequencing (NGS) technology has evolved rapidly in the last five years, leading to the generation of hundreds of millions of sequences (reads) in a single run. The number of generated reads varies between 1 million for long reads generated by Roche/454 sequencer (≈400 base pairs (bps)) and 2.4 billion for short reads generated by Illumina/Solexa and ABI/SOLIDTM sequencers (≈75 bps). The invention of the high-throughput sequencers has led to a significant cost reduction, e.g., a Megabase of DNA sequence costs only <DOLLAR/>0.1 [1].

Nevertheless, the large amount of generated data tells us almost nothing about the DNA, as stated by Flicek and Birney [2]. This is due to the lack of proper analysis tools and algorithms. Therefore, bioinformatics researchers started to think about new ways to efficiently handle and analyze this large amount of data.

One of the areas that attracted many researchers to work on is the alignment (mapping) of the generated sequences, i.e., the alignment of reads generated by NGS machines to a reference genome. Because, an efficient alignment of this large amount of reads with high accuracy is a crucial part in many applications’ workflow, such as genome resequencing [2], DNA methylation [3], RNA-Seq [4], ChIP sequencing, SNPs detection [5], genomic structural variants detection [6], and metagenomics [7]. Therefore, numerous tools have been developed to undertake this challenging task including MAQ [8], RMAP [9], GSNAP [10], Bowtie [11], Bowtie2 [12], BWA [13], SOAP2 [14], Mosaik [15], FANGS [16], SHRIMP [17], BFAST [18], MapReads, SOCS [19], PASS [20], mrFAST [6], mrsFAST [21], ZOOM [22], Slider [23], SliderII [24], RazerS [25], RazerS3 [26], and Novoalign [27]. Moreover, GPU-based tools have been developed to optimally map more reads such as SARUMAN [28] and SOAP3 [29]. However, due to using different mapping techniques, each tool provides different trade-offs between speed and quality of the mapping. For instance, the quality is often compromised in the following ways to reduce runtime: 

● Neglecting base quality score.

● Limiting the number of allowed mismatches.

● Disabling gapped alignment or limiting the gap length.

● Ignoring SNP information.

In most cases, it is unclear how such compromises affect the performance of newly developed tools in comparison to the state of the art ones. Therefore, many studies have been carried out to provide such comparisons. Some of the available studies were mainly focused on providing new tools (e.g., [10,13]). The remaining studies tried to provide a thorough comparison while each covering a different aspect (e.g., [30-34]).

For instance, Li and Homer [30] classified the tools into groups according to the used indexing technique and the features the tools support such as gapped alignment, long read alignment, and bisulfite-treated reads alignment. In other words, in that work, the main focus was classifying the tools into groups rather than evaluating their performance on various settings.

Similar to Li and Homer, Fronseca et al. [34] provided another classification study. However, they included more tools in the study, around 60 mappers, while being more focused on providing a comprehensive overview of the characteristics of the tools.

Ruffalo et al. [32] presented a comparison between Bowtie, BWA, Novoalign, SHRiMP, mrFAST, mrsFAST, and SOAP2. Unlike the above mentioned studies, Ruffalo et al. evaluated the accuracy of the tools in different settings. They defined a read to be correctly mapped if it maps to the correct location in the genome and has a quality score higher than or equal to the threshold. Accordingly, they evaluated the behavior of the tools while varying the sequencing error rate, indel size, and indel frequency. However, they used the default options of the mapping tools in most of the experiments. In addition, they considered small simulated data sets of 500,000 reads of length 50 bps while using an artificial genome of length 500Mbp and the Human genome of length 3Gbp as the reference genomes.

Another study was done by Holtgrewe et al. [31], where the focus was the sensitivity of the tools. They enumerated the possible matching intervals with a maximum distance *k* for each read. Afterwards, they evaluated the sensitivity of the mappers according to the number of intervals they detected. Holtgrewe et al. used the suggested sensitivity evaluation criteria to evaluate the performance of SOAP2, Bowtie, BWA, and Shrimp2 on both simulated and real datasets. However, they used small reference genomes (the S. cerevisiae genome of length 12 Mbp and the D. melanogaster genome of length 169 Mbp). In addition, the experiments were performed on small real data sets of 10,000 reads. For evaluating the performance of the tools on real data sets, Holtgrewe et al. used RazerS to detect the possible matching intervals. RazerS is a full sensitive mapper, hence it is a very slow mapper [21]. Therefore, scaling the suggested benchmark process for realistic whole genome mapping experiments with millions of reads is not practical. Nevertheless, after the initial submission of this work, RazerS3 [26] was published, thus, making a significant improvement in the running time of the evaluation process.

Schbath et al. [33] also focused on evaluating the sensitivity of the sequencing tools. They evaluated if a tool correctly reports a read as a unique or not. In addition, for non-unique reads, they evaluated if a tool detects all of the mapping locations. However, in their work, like many previous studies, the tools were used with default options, and they tested the tools with a very small read length of 40 bps. Additionally, the error model they used did not include indels and allowed only 3 mismatches.

Even though many studies have been published for evaluating short sequence mapping tools, the problem is still open and further perspectives were not tackled in the current studies. For instance, the above studies did not consider the effect of changing the default options and using the same options across the tools. In addition, some of the studies used small data sets (e.g., 10,00 and 500,000 reads) while using small reference genomes (e.g., 169Mbps and 500Mbps) [31,32]. Furthermore, they did not take the effect of input properties and algorithmic features into account. Here, input properties refer to the type of the reference genome and the properties of the reads including their length and source. Algorithmic features, on the other hand, pertain to the features provided by the mapping tool regarding its performance and utility. Therefore, there is still a need for a quantitative evaluation method to systematically compare mapping tools in multiple aspects. In this paper, we address this problem and present two different sets of experiments to evaluate and understand the strengths and weaknesses of each tool. The first set includes the benchmarking suite, consisting of tests that cover a variety of input properties and algorithmic features. These tests are applied on real RNA-Seq data and genomic resequencing synthetic data to verify the effectiveness of the benchmarking tests. The real data set consists of 1 million reads while the synthetic data sets consist of 1 million reads and 16 million reads. Additionally, we have used multiple genomes with sizes varying from 0.1 Gbps to 3.1 Gbps. The second set includes a use case experiment, namely, SNP calling, to understand the effects of mapping techniques on a real application.

Furthermore, we introduce a new, albeit simple, mathematical definition for the mapping correctness. We define a read to be correctly mapped if it is mapped while not violating the mapping criteria. This is in contrast to previous works where they define a read to be correctly mapped if it maps to its original genomic location. Clearly, if one knows “the original genomic location”, there is no need to map the reads. Hence, even though such a definition can be considered more biologically relevant, unfortunately this definition is neither sufficient nor computationally achievable. For instance, a read could be mapped to the original location with two mismatches (i.e., substitution error or SNP) while there might exist a mapping with an exact match to another location. If a tool does not have any *a-priori* information about the data, it would be impossible to choose the two mismatches location over the exact matching one. One can only hope that such tool can return “the original genomic location” when the user asks the tool to return all matching locations with two mismatches or less. Indeed, as later shown in the paper, our suggested definition is computationally more accurate than the naïve one. In addition, it complements other definitions such as the one suggested by Holtgrewe et al. [31].

To assess our work, we apply these tests on nine well known short sequence mapping tools, namely, Bowtie, Bowtie2, BWA, SOAP2, MAQ, RMAP, Novoalign, GSNAP, and mrFAST (mrsFAST). Unlike the other tools in this study, mrFAST (mrsFAST) is a full sensitive exact mapper that reports all the mapping locations. Therefore, comparing the mapping accuracy performance of mrFAST with the remaining tools is beneficial in further understanding the behavior of the different tools, even though comparing the execution time performance will not be fair. Moreover, we compare the performance of these tools with that of FANGS, a long read mapping tool, to show their effectiveness in handling long reads. The remaining tools were chosen according to the indexing techniques they use. Therefore, we can emphasize on the effect of the indexing technique on the performance. The experiments are carried out while using the same options for the tools, whenever possible.

The paper is organized as follows: in the next section, we briefly describe the sequence mapping problem, the mapping techniques used by the tools, and various evaluation criteria used to evaluate the performance of the tools including other definitions for mapping correctness. Then, we discuss how we designed the benchmarking suite and give a real application for the mapping problem. Finally, we present and explain the results for our benchmarking suite.

## Background

The exact matching of DNA sequences to a genome is a special case of the string matching problem. It requires incorporating the known properties or features of the DNA sequences and the sequencing technologies, thus, adding additional complexity to the mapping process. In this section, we first give a brief description of a set of features of DNA and sequencing technologies. Then, we explain how the tools used in this study work and support these features. Additionally, we describe the default options setup and show how divergent they are among the tools. Finally, we compare the evaluation criteria used in previous studies.

### Features

● *Seeding* represents the first few tens of base pairs of a read. The seed part of a read is expected to contain less erroneous characters due to the specifics of the NGS technologies. Therefore, the seeding property is mostly used to maximize performance and accuracy.

● *Base quality scores* provide a measure on correctness of each base in the read. The base quality score is assigned by a phred-like algorithm [35,36]. The score *Q* is equal to −10 log10(*e*), where *e* is the probability that the base is wrong. Some tools use the quality scores to decide mismatch locations. Others accept or reject the read based on the sum of the quality scores at mismatch positions.

● *Existence of indels* necessitates inserting or deleting nucleotides while mapping a sequence to a reference genome (gaps). The complexity of choosing a gap location increases with the read length. Therefore, some tools do not allow any gaps while others limit their locations and numbers.

● *Paired-end reads* result from sequencing both ends of a DNA molecule. Mapping paired-end reads increases the confidence in the mapping locations due to having an estimation of the distance between the two ends.

● *Color space read* is a read type generated by SOLiD sequencers. In this technology, overlapping pairs of letters are read and given a number (color) out of four numbers [17]. The reads can be converted into bases, however, performing the mapping in the color space has advantages in terms of error detection.

● *Splicing* refers to the process of cutting the RNA to remove the non-coding part (introns) and keeping only the coding part (exons) and joining them together. Therefore, when sequencing the RNA, a read might be located across exon-exon junctions. The process of mapping such reads back to the genome is hard due to the variability of the intron length. For instance, the intron length ranges between 250 and 65,130 nt in eukaryotic model organisms [37].

● *SNPs* are variations of a single nucleotide between members of the same species. SNPs are not mismatches. Therefore, their locations should be identified before mapping reads in order to correctly identify actual mismatch positions.

● *Bisulphite treatment* is a method used for the study of the methylation state of the DNA [3]. In bisulphite treated reads, each unmethylated cytosine is converted to uracil. Therefore, they require special handling in order not to misalign the reads.

### Tools’ description

For most of the existing tools (and for all the ones we consider), the mapping process starts by building an index for the reference genome or the reads. Then, the index is used to find the corresponding genomic positions for each read. There are many techniques used to build the index [30]. The two most common techniques are the followings: 

● Hash Tables: The hash based methods are divided into two types: hashing the reads and hashing the genome. In general, the main idea for both types is to build a hash table for subsequences of the reads/genome. The key of each entry is a subsequence while the value is a list of positions where the subsequence can be found. Hashing based tools include the following tools:

**GSNAP**[10] is a genome indexing tool. The hash table is built by dividing the reference genome into overlapping oligomers of length 12 sampled every 3 nucleotides. The mapping phase works by first dividing the read into smaller substrings, finding candidate regions for each substring, and finally combining the regions for all of the substrings to generate the final results. GSNAP was mainly designed to detect complex variants and splicing in individual reads. However, in this study, GSNAP is only used as a mapper to evaluate its efficiency.

**Novoalign**[27] is a genome indexing tool. Similar to GSNAP, the hash table is built by dividing the reads into overlapping oligomers. The mapping phase uses the Needleman-Wunsch algorithm with affine gap penalties to find the global optimum alignment.

**mrFAST and mrsFAST**[6,21] are genome indexing tools. They build a collision free hash table to index *k*-mers of the genome. mrFAST and mrsFAST are both developed with the same method, however, the former supports gaps and mismatches while the latter supports only mismatches to run faster. Therefore, in the following, we will use mrsFAST for experiments that do not allow gaps and mrFAST for experiments that allow gaps. Unlike the other tools, mrFAST and mrsFAST report all of the available mapping locations for a read. This is important in many applications such as structural variants detection.

**FANGS**[16] is a genome indexing tool. In contrary to the other tools, it is designed to handle the long reads generated by the 454 sequencer.

**MAQ**[8] is a read indexing tool. The algorithm works by first constructing multiple hash tables for the reads. Then, the reference genome is scanned against the tables to find the mapping locations.

**RMAP**[9] is a read indexing tool. Similar to MAQ, RMAP pre-processes the reads to build the hash table, then the reference genome is scanned against the hash table to extract the mapping locations.

Most of the newly developed tools are based on indexing the genome. Nevertheless, MAQ and RMAP are included in this study to investigate the effectiveness of our benchmarking tests on evaluating read indexing based tools. In addition, we investigate if there is any potential for the read indexing technique to be used in new tools.

● Burrows-Wheeler Transform (BWT):

**BWT**[38] is an efficient data indexing technique that maintains a relatively small memory footprint when searching through a given data block. BWT was extended by Ferragina and Manzini [39] to a newer data structure, named FM-index, to support exact matching. By transforming the genome into an FM-index, the lookup performance of the algorithm improves for the cases where a single read matches multiple locations in the genome. However, the improved performance comes with a significantly large index build up time compared to hash tables.

BWT based tools include the following:

**Bowtie**[11] starts by building an FM-index for the reference genome and then uses the modified Ferragina and Manzini [39] matching algorithm to find the mapping location. There are two main versions of Bowtie namely Bowtie and Bowtie 2. Bowtie 2 is mainly designed to handle reads longer than 50 bps. Additionally, Bowtie 2 supports features not handled by Bowtie. It was noticed that both versions had different performance in the experiments. Therefore, both versions are included in this study.

**BWA**[13] is another BWT based tool. The BWA tool uses the Ferragina and Manzini [39] matching algorithm to find exact matches, similar to Bowtie. To find inexact matches, the authors provided a new backtracking algorithm that searches for matches between substring of the reference genome and the query within a certain defined distance.

**SOAP2**[14] works differently than the other BWT based tools. It uses the BWT and the hash table techniques to index the reference genome in order to speed up the exact matching process. On the other hand, it applies a “split-read strategy”, i.e., splits the read into fragments based on the number of mismatches, to find inexact matches.

In addition to providing different mapping techniques, each tool handles only a subset of the DNA sequences and the sequencing technologies features. Moreover, there are differences in the way the features are handled, which are summarized in Table 1. For instance, BWA, SOAP, and GSNAP accept or reject an alignment based on counting the number of mismatches between the read and the corresponding genomic position. On the other hand, Bowtie, MAQ, and Novoalign use a quality threshold (i.e., alignment score) to perform the same function. The quality threshold is different from the mapping quality. The former is the probability of the occurrence of the read sequence given an alignment location while the latter is the Bayesian posterior probability for the correctness of the alignment location calculated from all of the alignments found for the read.

**Table 1:** Features supported by the tools

	**Bowtie**	**Bowtie2**	**BWA**	**SOAP2**	**MAQ**	**RMAP**	**GSNAP**	**FANGS**	**Novoalign**	**mrFAST**	**mrsFAST**
Seed mm.	Up to 3		Any	Up to 2	Any	Any					
Non-seed mm.	QS	AS	Count	Count	QS	Count	Count	Count	QS	Count	Count
Var. seed len.	> 5		Any	> 28							
Mapping qual.		Yes	Yes		Yes				Yes		
Gapped align.		Yes	Yes	PE	PE		Yes	Yes	Yes	Yes	
Colorspace	Yes		Yes		Yes				Yes		
Splicing							Yes				
SNP tolerance							Yes				
Bisulphite reads						Yes	Yes		Yes	Yes	

PE: paired-end only, mm.: mismatches, QS: base quality score, count: total count of mismatches in the read, AS: alignment score, and empty cells mean not supported.

In some cases, the features are partially supported. For example, SOAP2 supports gapped alignment only for paired end reads, while BWA limits the gap size. Therefore, considering only one of the above features when comparing between the tools would lead to under- or over-estimation of the tools’ performance.

### Default options of the tested tools

In general, using a tool’s default options yields a good performance while maintaining a good output quality. Most users use the tools with the default options or only tweak some of them. Therefore, it is important to understand the effect of using these options and the kind of compromises made while using them. For the nine tools considered in this paper, the most crucial default options are the following: 

● Maximum number of mismatches in the seed: the seed based tools use a default value of 2.

● Maximum number of mismatches in the read: Bowtie2, BWA, and GSNAP determine the number of mismatches based on the read length. It is 10 for RMAP, 2 for mrsFAST, and 5 for SOAP2, FANGS, and mrFAST.

● Seed length: It is 28 for MAQ, 32 for RMAP, and 28 for Bowtie. BWA disables seeding while SOAP2 considers the whole read as the seed.

● Quality threshold: It is equal to 70 for MAQ and Bowtie while it depends on the read length and the genome size for Novoalign.

● Splicing: This option is enabled for GSNAP.

● Gapped alignment: It is enabled for Bowtie2, GSNAP, BWA, Novoalign and MAQ while it is disabled for SOAP2.

● Minimum and maximum insert sizes for paired-end mapping: The insert size represents the distance between the two ends. The values used for the minimum and the maximum insert sizes are 0 and 250 for Bowtie and MAQ, 0 and 500 for BWA and Bowtie2, 400 and 500 for SOAP2, and 100 and 400 for RMAP. mrFAST and mrsFAST do not have default values for max and min insert sizes.

Indeed, as will be shown in the results’ section, having different default values lead to different results for the same data set. Hence, using the same values when comparing between the tools is important.

### Evaluation criteria

In general, the performance of the tools is evaluated by considering three aspects, namely, the *throughput* or the *running time*, the *memory footprint*, and the *mapping percentage*. The *throughput* is the number of base pairs mapped per second (bps/sec) while the *memory footprint* is the required memory by the tool to store/process the read/genome index. The *mapping percentage* is the percentage of reads each tool maps.

**Figure 1:**
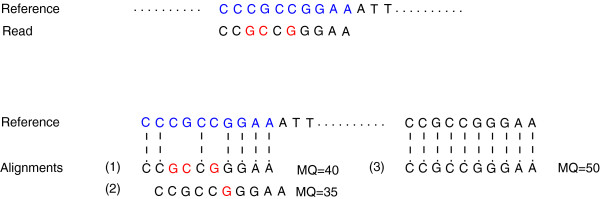
**Evaluation criteria.** An example showing how the different evaluation criteria work. In the upper part of the figure, the sequence in blue is the original genomic position where the simulated read was extracted from. After applying sequencing errors, the read does not exactly match to the original location (3 mismatches). In the lower part of the figure, three possible alignment locations for the read are shown with their mapping quality score (MQ). The naïve criterion would only consider the alignment (1) as the correct alignment. For Ruffalo et al. [32] criterion, if the used threshold is 30, then (1) is *correctly mapped* while (2) and (3) are *incorrectly mapped-strict*. On the other hand, if the threshold is 40, then (3) is considered as *incorrectly mapped relaxed*. Holtgrewe et al. [31] criterion in the oracle mode would detect (1) and (2) and consider them *correctly mapped* while (3) would be considered as *incorrectly mapped*.

The *mapping percentage* is further divided into a *correctly mapped* reads part and an *error* (false positives) part. There have been many definitions suggested for the *error* in previous studies. For instance, for the simulated reads, the naïve and most used definition for *error* is the percentage of reads mapped to the incorrect location (i.e., a location other than the genomic location the read was originally extracted from) [10,13]. Clearly, this definition is neither sufficient nor computationally correct. Figure 1 gives an example explaining the drawbacks of this definition. After applying sequencing errors, the read does not exactly match the original genomic location. Since the tools do not have any *a-priori* information for the data, it would be impossible to choose the two mismatches location as the best mapping location over the exact matching one. Therefore, the naïve criteria would judge the tool as incorrectly mapping the read if the tool returned either alignment (2) or (3) while in fact it picked a more accurate matching.

The naïve definition for the *error* was further modified by Ruffalo et al. [32] to develop a more concrete definition. The authors incorporated the mapping quality information such that a read is *correctly mapped* if it is mapped to the original genomic location while having a mapping quality greater than a certain threshold. They further categorized the incorrectly mapped reads into *incorrectly mapped-strict* and *incorrectly mapped-relaxed*. The *incorrectly mapped-strict* are the reads that were mapped with a quality higher than the threshold while not mapped to the original genomic location. On the other hand, the *incorrectly mapped-relaxed* are the reads that were mapped to an incorrect location with a quality higher than the threshold and there is no correct mapping for the read with a mapping quality higher than the threshold. As an example, in Figure 1, if the used threshold is 30, then the read would be considered *correctly mapped* if the tool returned alignment (1) while it would be considered as *incorrectly mapped-strict* if the tool returned either alignment (2) or (3). On the other hand, if the used threshold is 40, a read would be *incorrectly mapped-relaxed* if the tool returned alignment (3). Indeed, this is a valuable evaluation criterion, however, many tools, such as SOAP2, RMAP, and BWA, do not use quality scores in the mapping phase. In addition, not all of the tools report the mapping quality.

Another definition was introduced by Holtgrewe et al. [31]. Unlike the previous works, the authors tried to find a gold standard for each read, where a gold standard refers to all of the possible matching intervals for each read with a maximum distance *k* from the read. To enumerate all of the possible matching intervals, the authors used RazerS to detect the initial seed location for each interval. Afterwards, they developed a method to find the boundary of the interval centered at the seed and with a maximum distance *k* from the read. They named the suggested evaluation method Rabema. As an example, a possible interval with *k*=3 would contain alignment (1) and (2) in Figure 1. Accordingly, Holtgrewe et al. defined the *false negatives* as the intervals missed by the mapper and the *false positives* as the intervals returned by the mapper and not included in the gold standard. However, consisting of seed detection phase and enumeration phase while depending on RazerS to return seed locations for the matching intervals makes Rabema impractical to apply on large genomes and long read lengths, e.g., RazerS took 25 hours to map 1 million reads of length 100 to the Human genome while doubling the running time when increasing the read length from 75 to 100 [21]. Therefore, Holtgrewe et al. suggested another mode, an *oracle mode*, which makes use of the original location of simulated reads. The *oracle mode* uses the original location as the seed location instead of using RazerS to detect the initial seed locations. However, this method is only suitable in case of *a-priori* knowledge that the possible mapping locations for a read are around the simulated location (e.g., alignment (3) in Figure 1 would be missed in the *oracle mode*). Nevertheless, after the initial submission of this work, RazerS3 [26] was published; making a significant improvement in Rabema running time and elevating the slowness problem. Even though the suggested definition for a gold standard quantitatively estimates the sensitivity for each mapper, it suffers from a couple of drawbacks. First, the definition does not take into consideration whether the alignments are violating the mapping criterion for the mapper or not. For instance, in Figure 1, the sensitivity of the mapper would increase if it detected alignments (1), (2), and (3). However, if the mapping criterion for the mapper is to allow a maximum of two mismatches, then alignment (1) should have not been detected by the mapper and should be considered as a wrong alignment or error. Second, quality aware based tools, such as Bowtie, MAQ, and Novoalign, would be incorrectly evaluated by Rabema since they use the quality threshold to accept or reject a read instead of calculating the edit or hamming distance. Therefore, they might map a read with more mismatches than the limit allowed by Rabema.

## Methods

### Benchmark design

In this section, we present the features covered by our benchmarking suite. In addition, we explain how they were previously addressed by the tools we mention in this paper. However, two algorithmic features, namely SNPs and Splicing awareness, are not presented in the results section due to being supported only by one tool. The tests are categorized as follows: 

● Mapping options

**Quality threshold**: MAQ, Bowtie, and Novoalign use the quality threshold to determine the number of allowed mismatches. Therefore, setting a quality threshold is similar to explicitly setting the number of mismatches. However, there is no hard limit on the actual number of mismatches. The impact of varying the quality threshold while finding a mapping between the quality threshold and the number of mismatches has not been studied before.

**Number of mismatches**: Changing the number of allowed mismatches affects the percentage of mapped reads. This effect was studied in [10], however, the mismatches were generated uniformly on the genome which does not mimic real mismatches distribution.

**Seed length**: Seeding-based tools impose limits on the number of mismatches in the seed part. As a result, increasing or decreasing the length of the seed part affects the percentage of mapped reads. The effect of the seed length has not been studied in details before.

● Input properties

**Read length**: The read length varies between 30bps for ABI’s SOLiD and Illumina’s Solexa sequencers up to 500 bps for Roche’s 454. Therefore, the impact of read length should be considered for throughput evaluation. Even though the effect of the read length was explored in several studies, the default options were usually used leading to incomparable trade-offs.

**Paired-end reads**: Mapping paired reads requires the mapping of both ends within a maximum distance between them. Hence, it adds a constraint when finding the corresponding genomic locations.

**Genome type**: The efficiency of most algorithms are tested by using the Human genome as the reference. However, each genome has its own properties such as the percentage of repeated regions and ambiguous characters. Therefore, using a single genome does not reveal the effect of these properties. To the best of our knowledge, BWA [13] was the only tool to test its performance on a large genome other than the Human.

● Algorithmic features

**Gapped alignment**: is important for variant discovery due to the ability to detect indel polymorphism [30]. Bowtie2, GSNAP, Novoalign, BWA, and mrFAST are the only tools to support it for single-end reads while the remaining tools support it for paired-end only. However, from the results provided by the previous studies, it is not obvious how gapped alignment affects the performance of the tools in comparison to allowing only mismatches.

**SNP awareness**: Incorporating SNP information into mapping allows considering minor alleles as matches rather than mismatches. Currently, this feature is provided only by GSNAP. It was shown in [10] that integrating SNP information affected around 8% of the reads and allowed mapping 0.4% of unmapped reads.

**Splicing awareness**: Reads located across exon-exon junctions would be wrongly aligned using standard alignment algorithms. Splicing awareness is only required for certain types of data such as RNA-Seq data. The only tool that currently supports splicing while performing the mapping phase is GSNAP. It was shown in [10] that the alignment yield increased by 8-9% when splicing detection based on known splice junctions was introduced. However, there was only 0.3-0.6% increase in case of detecting novel splice junctions.

● Scalability

The scalability of the mapping tools may be different under different parallel settings. Many tools support multithreading, which is expected to yield linearly increasing speedup with the increase in the number of CPU cores. On the other hand, using multiprocessing is more general and may improve the throughput even for tools that do not support multithreading (e.g., MAQ and RMAP), where multiprocessing refers to using more than one process in a distributed memory fashion while communicating through a message passing interface.

● Accuracy evaluation

Each tool is expected to map a set of reads based on its mapping criteria. However, a subset of the reads might not be mapped (i.e., *false negatives*) due to using heuristics in the mapping algorithm or the default options limitations. Moreover, some of the tools map a subset of these reads while violating the mapping criteria.

● Rabema evaluation

Rabema benchmark enumerates all of the possible matching locations. Then, it evaluates whether the tool detected the possible matching locations with the specified error rate or not. Therefore, Rabema evaluation is a valuable one and helps in adding another perspective when comparing between the tools.

### Usecase: SNP calling

SNP calling is the process of detecting genetic variations in a given genome. The genetic variations contribute to the generation of different phenotypes for the same gene, leading to increasing the risk of having complex diseases. Therefore, the discovery of SNPs is a very important process that needs to be done accurately. Many tools have been developed to detect SNPs including ssahaSNP [40] and SNPdetector [41]. These tools were developed to analyze the DNA sequences generated using either the Sanger or the direct PCR amplification methods. However, with the development of the next generation sequencing technology, new tools are required to analyze the new data [42]. The developed new tools work by first mapping sequences to a reference genome, then using statistical analysis methods to extract SNPs [42] after filtering out low-quality mismatches. Therefore, accurately mapping the reads to the reference genome is a very crucial task in the SNP calling pipeline.

## Results and discussion

In this section, we present the results from our benchmarking tests. The experiments were performed on a cluster of quad-core AMD Opteron CPUs at 2.4 GHz with 32 GB of RAM. We used SOAP2 v2.20, Bowtie v0.12.6 and v2.0.0-beta5, BWA v0.5.0, MAQ v0.7.0, RMAP v2.05.0, FANGS v0.2.3, GSNAP v2010-07-27, Novoalign v2.07.0, and mrFAST and mrsFAST v2.5.0.4.

**Performance evaluation**: The performance is evaluated by considering two factors, namely, the mapping percentage and the throughput. The mapping percentage is the percentage of reads each tool maps while the throughput is the number of mapped base pairs per second (bps/sec). The throughput is calculated by dividing the number of reads mapped over the running time. For genome indexing based tools, the running time includes only the matching time while it includes the indexing and matching time for read indexing based tools. However, the running time for mrsFAST includes also the indexing time even though it is a genome indexing based tool. This is due to the dependence of the sensitivity of mrsFAST on the window size used in the indexing phase. Therefore, the index is rebuilt in most of the experiments to maintain a full sensitivity for mrsFAST.

In addition, the mapping percentage is further divided into the following: 

● *Correctly mapped reads*: The percentage of reads mapped within the mapping criteria.

● *Error*: The percentage of reads mapped while violating the mapping criteria. As shown in the background section, this definition provides another evaluation perspective that was not covered by older definitions.

● *Amb*: The percentage of reads mapped to more than one location with the same number of mismatches. Most of the tools can return more than one mapping location for *Amb* reads if desired. However, RMAP only reports the number of *Amb* reads while not providing any information regarding the mapping location and the number of mismatches. Therefore, we will not be able to report the mismatches distribution for the RMAP reported *Amb* reads.

**Data sets**: We evaluated the tools on two types of data sets, namely, synthetic data and real data. The synthetic data set mimics reads generated from genomic sequencing while the real data set is for RNA-Seq. The data sets are further generated as follows: 

● *Synthetic data*: There is a number of tools available to extract synthetic, Fastq format, data sets from a reference genome including wgsim[43], dwgsim[44], Mason[45], and ART[46]. wgsim generates reads with uniform error distribution while dwgsim provides a uniformly increasing/decreasing error rate. On the other hand, Mason and ART mimic the error rates for Illumina and 454 sequencers. In this study, we are using wgsim and ART to generate the synthetic data from the Human genome. wgsim helps in providing a fair comparison between the tools by using a uniform error distribution model resulting in the same quality score for each base. Therefore, all of the tools can be allowed exactly the same number of mismatches regardless of the technique used to set the maximum number. For wgsim, the reads were generated with 0.09% SNP mutation rate, 0.01% indel mutation rate, 2% uniform sequencing base error rate, and with a maximum insert size of 500, which are the same parameters used in [13]. Additionally, Dohm et al. [47] showed that the sequencing error rate for Illumina changes between 0.3% for the beginning of the read and 3.8% at the end of the read. Therefore, a uniform sequencing error rate of 2% is acceptable. Moreover, according to the error rates and indels rate used by the Mason simulator [45], an indel rate of 0.01% is acceptable. We determined the number of reads to generate using wgsim based on the used tool and the experiment. On the other hand, ART does not explicitly allow the user to choose the number of generated reads. ART generates reads that cover the whole genome with a given coverage level. Therefore, to manage generating 1 million reads, we used ART to generate reads that cover the whole genome with 1x coverage. Then, we randomly selected 1 million reads from the output reads.

● To make sure that the results are not affected by different wgsim runs, we generated 13 different wgsim data sets and ran a sample of the tools independently on each data set. The sample included BWA, GSNAP, Bowtie, Bowtie2, and SOAP2. We found that the maximum standard deviation from the average was 0.03 (results are not included). Since there is no significant change between the runs, we will only carry each experiment once on a single data set.

● *Real data*: There are many types of real data sets such as RNA-Seq data, Chip-Seq data, and DNA sequences that are used in different applications. It is important in our evaluation process to choose the right data set type to better evaluate the applicability of the tools in the different applications. Therefore, we prefer to use RNA-Seq data sets as they are used in many applications including SNP and alternative splicing detection. The used data set consists of 1 million reads generated by Illumina sequencer after isolating mRNA from the Spretus mouse colon tissues. The mouse genome version mm9 was used as the reference genome. Indeed, as will be shown, the tools have similar behavior on both the mouse and the human genomes. Therefore, there is no contradiction in using the human genome for generating the synthetic data while the mouse genome is used for the real ones.

**Figure 2:**
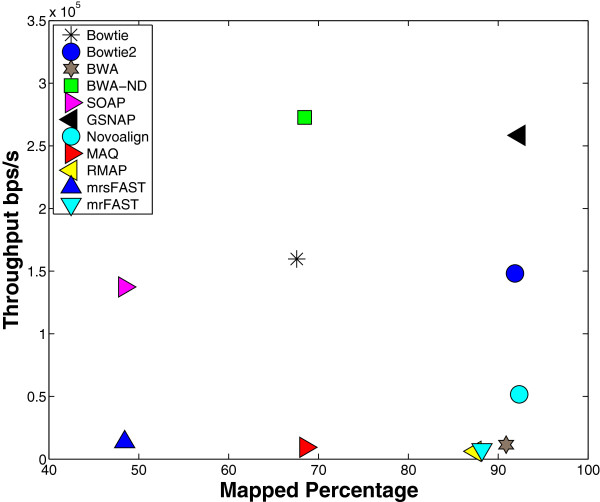
**Default options effect using ****wgsim****.** Mapping 1 million reads of length 125 extracted from the Human genome using wgsim. Each tool was allowed to use its own default options. BWA-ND refers to BWA’s results while using Bowtie’s default options which are 2 mismatches in the seed, 3 mismatches in the whole read, and no gapped alignment.

**Figure 3:**
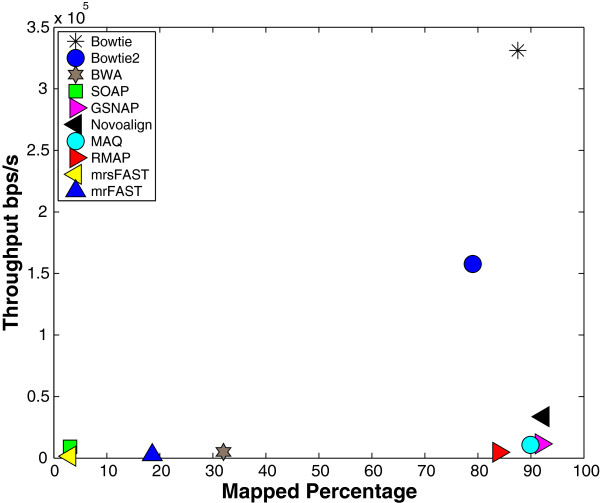
**Default options effect using ART.** Mapping 1 million reads of length 100 extracted from the Human genome using ART. Each tool was allowed to use its own default options.

First, we present the effect of the default options. The results for this experiment are given in Figures 2 and 3. Figure 2 shows the results when using wgsim to generate the synthetic data while Figure 3 shows the results using ART. As stated previously, tools try to use the options that yield a good performance while maintaining a good output quality. For instance, as shown in Figure 2, Bowtie achieves a throughput of around 1.6·10^5^bps/s at the expense of mapping only 67.58% of the reads. On the other hand, BWA maps 91% of the reads at the expense of having a throughput of 0.1·10^5^ bps/s. Additionally, SOAP and mrsFAST (Figures 2 and 3) would look like that they provide the smallest mapping. However, percentage they are only allowing 2 mismatches while other tools such as mrFAST and GSNAP are allowing more than 5 mismatches. Therefore, using only the default options to build our conclusions would be misleading. Indeed, further experiments show that BWA obtains a high throughput when allowed to use the same options as Bowtie (see BWA-ND in Figure 2). Moreover, BWA achieves a higher throughput than Bowtie in other experiments. Therefore, it is important to use the same options to truly understand how the tools behave.

In the remaining experiments, unless otherwise stated, the number of mismatches in the seed and in the whole read are fixed to 2 and 5, respectively, while the quality threshold is kept at 100. The minimum and maximum insert sizes allowed are 0 and 500, respectively. In addition, the splicing, SNPs, and gapped alignment options are disabled, unless otherwise stated. For the number of reported hits, tools are only allowed to report one location except for mrsFAST that does not have this option and report all of the mapping locations. The default values are used for the remaining options.

### Mapping options

**Quality threshold** is one of the two main metrics used for mismatch tolerance. The other main metric is the explicit specification of the number of mismatches. To compare fairly between the tools, a relationship between the two metrics should be found, which is the main target of this experiment. In this experiment, wgsim is used to generate the data set instead of using ART or a real one. The different base quality scores in real data cause quality threshold based tools to allow more mismatches than the other tools. For instance, when allowing a quality threshold of 70 and 5 mismatches for the remaining tools, Bowtie and MAQ map reads with up to 10 mismatches while the other tools are limited to 5 (results are not included). Therefore, MAQ and Bowtie had a mapping percentage larger than the other tools, hence, the comparison is not fair. Nevertheless, in the following, we show how the quality threshold can be used to mimic the behavior of the explicit specification of the number of mismatches.

**Figure 4:**
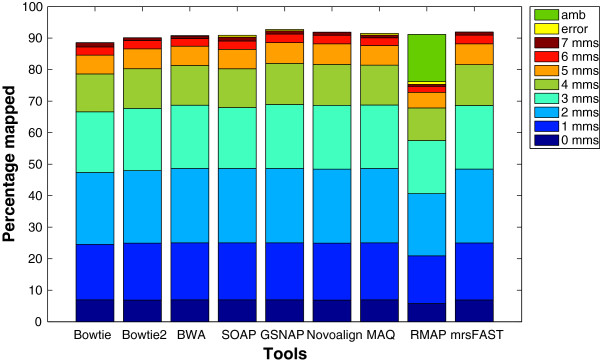
**Quality threshold vs. number of mismatches.** Mapping 1 million reads of length 125 extracted using wgsim from the Human genome while allowing up to 7 mismatches and a quality threshold of 140. The *error* is 0.6% for SOAP2 and MAQ and 0.45% for GSNAP.

For wgsim generated synthetic data, quality thresholds of 60, 80, 100, 120, and 140 should correspond to 3, 4, 5, 6, and 7 mismatches. To assess our conclusion, we designed an experiment where all tools were allowed a maximum of 7 mismatches while using a quality threshold of 140. Figure 4 shows that the tools map the reads with the same maximum number of mismatches while having similar mapping rates. However, the differences in the mapping rates are due to the pruning of the search space done by the default options for some of the tools. In addition, other tools incorrectly mapped some of the reads causing an increase in the mapping percentage. For instance, 0.6% of reported hits for MAQ and SOAP2 are considered as *error* (i.e., reads mapped while violating the mapping criteria) while Bowtie’s default options limit the allowed number of backtracks to find mismatches. On the other hand, GSNAP and mrsFAST map around 92% of the reads even though GSNAP reports *error* hits. This is due to being non-seed based tools, thus allowing more mismatches to be found in the first few base pairs. Additionally, mrsFAST is a full sensitive mapper, therefore, it can detect reads missed by other tools.

**Figure 5:**
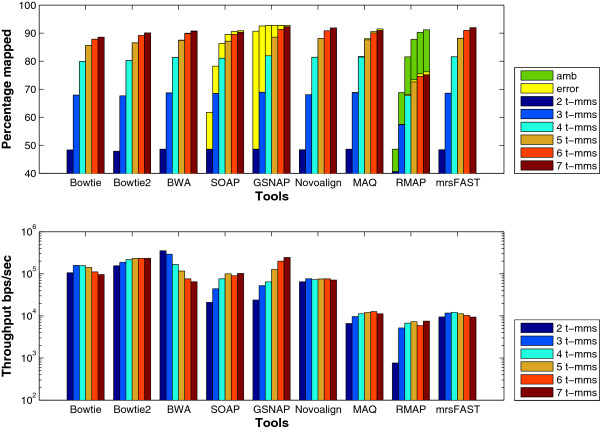
**Effect of changing the number of mismatches using a synthetic data set extracted using ****wgsim****.** Comparing the different tools while changing the total mismatches from 2 to 7. T-mms stands for the maximum allowed mismatches. A data set of 1 million reads of length 125 extracted from the Human genome using wgsim was used in this experiment.

**Number of mismatches**: Not only does the number of mismatches affect the percentage of mapped reads, but also affects the throughput. In particular, the mapping percentage increases nonlinearly with the number of mismatches. Figure 5 shows the effect of the number of mismatches in more details using a wgsim generated data set. There is a 20% increase in the percentage of mapped reads when allowing 3 mismatches instead of 2. On the other hand, there is less than 0.7% increase when allowing 7 mismatches instead of 6. In addition, the *error* percentage decreases for large number of mismatches. For instance, SOAP2’s *error* percentage is 21% when allowing 2 mismatches while it is reduced to 1% when allowing 6 mismatches. Additionally, mrsFAST mapped around 0.1-0.5% more reads than the remaining tools since it is a full sensitive mapper. From the throughput point of view, the tools behave differently. For instance, Bowtie, MAQ, RMAP, and mrsFAST are able to maintain almost the same throughput while the throughput increases for SOAP2 and GSNAP and decreases for BWA. The degradation in BWA’s performance is due to exceeding the default number of mismatches leading to excessive backtracking to find mismatch locations.

**Figure 6:**
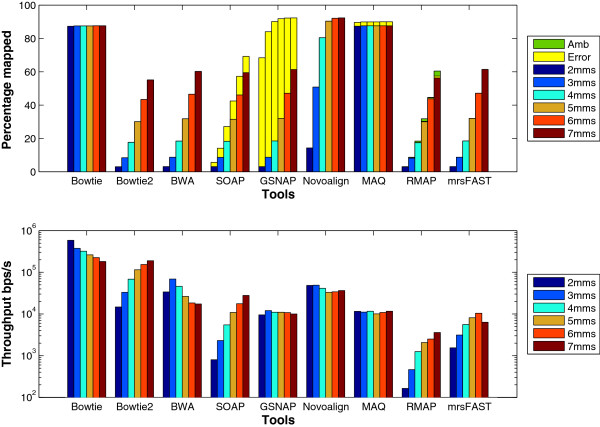
**Effect of changing the number of mismatches using a synthetic data set extracted using ****ART****.** Comparing the different tools while changing the total mismatches from 2 to 7. T-mms stands for the maximum allowed mismatches. A data set of 1 million reads of length 100 extracted from the Human genome using ART was used in this experiment.

Additionally, we used a data set of 1 million reads of length 100 generated by ART to evaluate the tools. The results for this experiment are shown in Figure 6. Similar to the wgsim results, the increase in the percentage of mapped reads when allowing 2 mismatches instead of 3 is larger than the increase when allowing 7 mismatches instead of 6. Unlike wgsim results, Bowtie maintains a higher throughput than Bowtie2 for the different number of mismatches. This is due to the difference in the read length between wgism and ART data sets (100 for ART instead of 125). Moreover, Bowtie uses the quality threshold while Bowtie2 does not.

**Figure 7:**
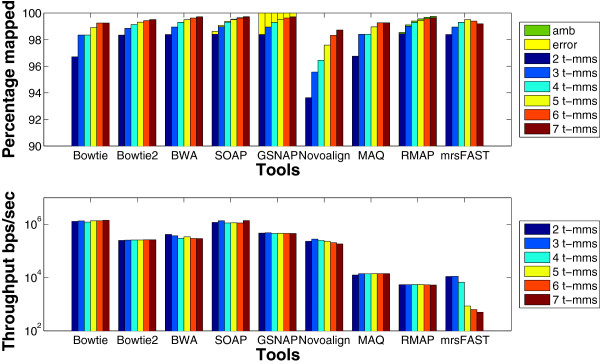
**Effect of changing the number of mismatches using a real data set.** Comparing the different tools while changing the total mismatches from 2 to 7. T-mms stands for the maximum allowed mismatches. A real mRNA data set of 1 million reads of length 51 bps extracted from the Spretus mouse strain and mapped against the mouse genome version mm9 was used in this experiment.

To further understand the behavior of the tools, the same set of experiments is applied on the mouse mRNA real data set. The results given in Figure 7 show that the *error* percentage for GSNAP still decreases for large number of mismatches. In addition, there is a small reduction in BWA’s throughput for large number of mismatches. Interestingly, the throughput for mrsFAST is different between the synthetic data and the real data. In the synthetic data set, mrsFAST’s throughput is higher than RMAP while maintaining the same throughput across the different number of mismatches. On the other hand, on the real data, the throughput decreases with the increase in the number of mismatches. In addition, there is a 7x reduction in the throughput between 4 t-mms and 5 t-mms. To maintain a full sensitivity for small read lengths while allowing a large number of mismatches, mrsFAST requires the use of a small window size when building the index (window size of 8 for 5 t-mms instead of 10 for 4 t-mms). The smaller the window size, the longer it takes to process the index. Additionally, there is a limit on the window size (min 8 and max 14). Therefore, mrsFAST starts to lose its sensitivity for detecting mapping locations for 6 and 7 t-mms.

**Figure 8:**
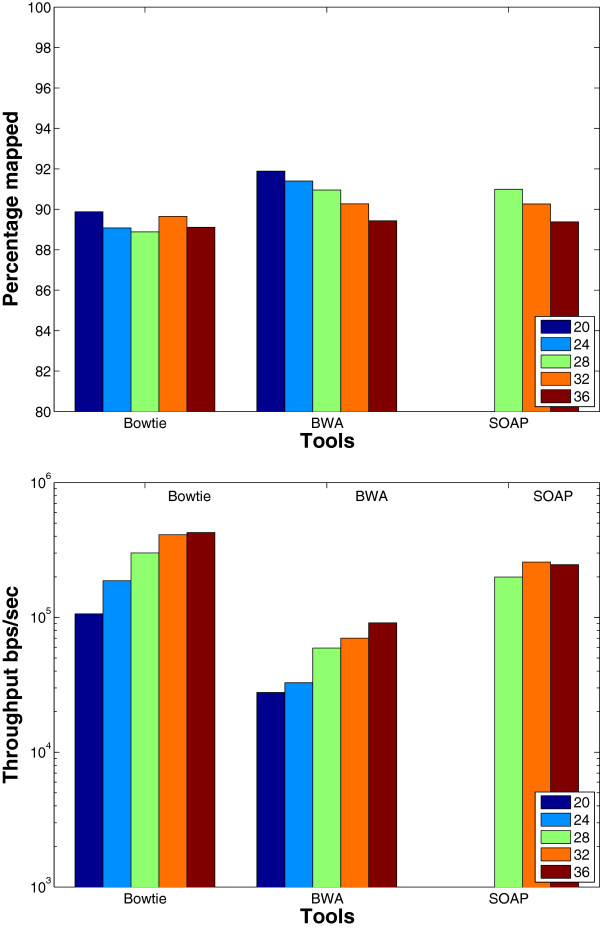
**Effect of changing the seed length using a synthetic data set.** The effect of changing the seed length on the BWT based tools. The tools were used to map 16 million reads of length 70 bps on the Human genome. SOAP2 does not support seed length < 28.

**Seed length**: Theoretically, when fixing the number of allowed mismatches in the seed and in the whole read, changing the seed length affects the mapping results. Specifically, a shorter seed allows more mismatches in the remaining part of the read to be found. Therefore, the percentage of mapped reads would increase even though the throughput would decrease. On the other hand, having a longer seed would result in pruning some parts of the search tree as soon as possible; leading to throughput improvement. The aim of this experiment is to study this trade off. As shown in the results given in Figure 8 using a wgism data set, the tools behave as expected. However, there are some exceptions. For instance, when increasing the seed length from 32 to 36 the percentage of mapped reads for SOAP2 and Bowtie decreases, however the throughput is not affected. In addition, there is a 0.8% increase in the percentage of mapped reads for Bowtie when increasing the seed length from 28 to 32. This behavior is due to the backtracking property that stops once a certain limit is reached. Therefore, as a result of having less erroneous bases in the seed part, Bowtie can continue more in the depth first search without exceeding the backtracking limit.

**Figure 9:**
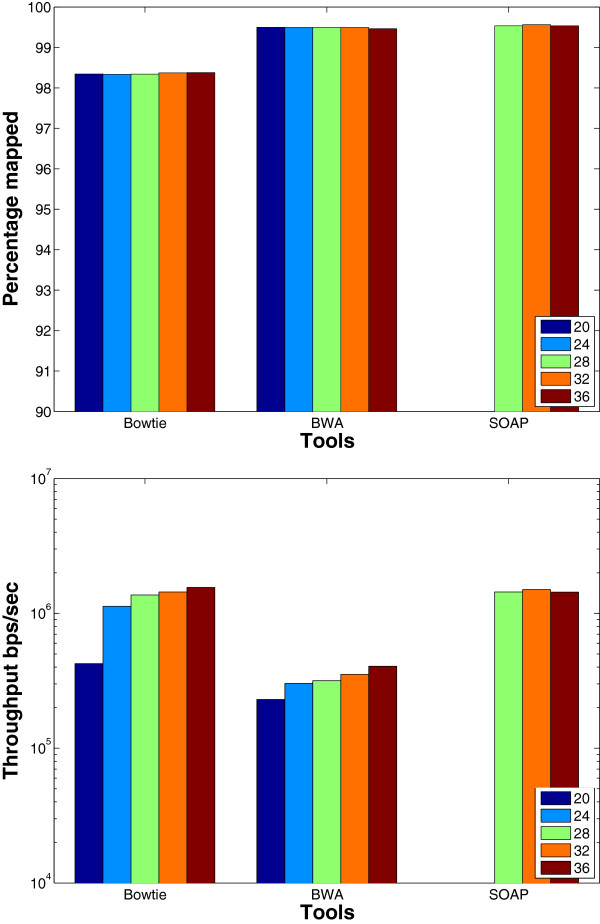
**Effect of changing the seed length using a real data set.** The effect of changing the seed length on the BWT based tools. The tools were used to map real mRNA data set of 1 million reads of length 51 bps extracted from the Spretus mouse strain on the mouse genome version mm9. SOAP2 does not support seed length < 28.

We also carried out the same experiment on the real mouse mRNA data set. The results given in Figure 9 show that the same behavior for Bowtie is still obtained on real data. However, Bowtie has only 0.01% increase when increasing the seed length from 28 to 32 instead of the 0.8% obtained in synthetic data.

### Input properties

**Figure 10:**
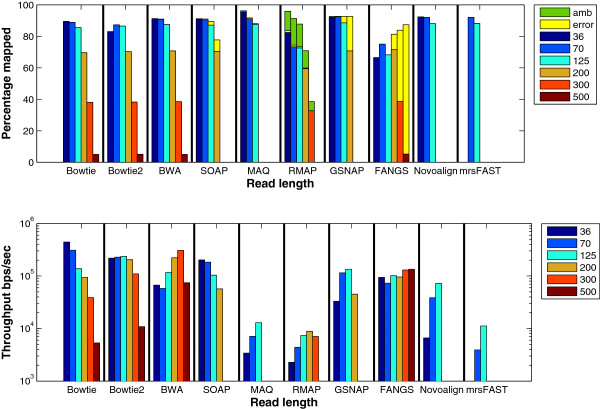
**Effect of changing the read length using a synthetic data set extracted using ****wgsim****.** The effect of changing the read length from 36 to 500. The reads were extracted from the Human genome. RMAP and MAQ are slower than the other tools. Therefore, 1 million reads were used to test MAQ and RMAP while 16 million reads were used for the remaining ones.

**Read length**: Longer reads tend to have more mismatches beside requiring more time to be fully mapped [48]. In general, for a fixed number of mismatches, increasing the read length decreases the percentage of mapped reads. Therefore, the aim of this experiment is to understand the read length effect. The results in Figure 10 show that the mapping percentage decreases with the increase in the read length while the *error* percentage increases. As an example, 95% of FANGS’ output for read length 500 is *error* compared to 12% of its output for read length 200. This is due to the increase of the erroneous bases with the increase of the read length. Therefore, it becomes harder to map the reads with the specified mapping criteria. In addition, Bowtie, Bowtie2, and BWA were the only short sequence mapping tools that managed to map long reads. In particular, the max read length was 128 for MAQ, 300 for RMAP, and 200 for GSNAP, 199 for mrsFAST, while SOAP2 took more than 24 hours to map the reads with length 300 and hence not reported. On the other hand, mrsFAST’s run on read length 36 was suddenly terminated. This is probably due to the small read length and the large number of mismatches. From the throughput point of view, tools do not maintain the same behavior. For instance, the throughput of Bowtie and SOAP2 decreases for long read lengths. This is due to the backtracking property and the split strategy [14] used by Bowtie and SOAP2, respectively, to find inexact matches. Moreover, Bowtie is better than Bowtie2 for read lengths 36 and 70. On the other hand, even though the throughput of BWA and GSNAP increases with the read length, it starts to decrease for read length 500 and 200, respectively. GSNAP works by combining position lists to create candidate mapping regions. Therefore, for long reads, the throughput decreases due to the increase in the work needed to generate and combine position lists. For mrsFAST, the throughput increases with the read length since the available mapping locations for a read are less for longer reads in comparison to small ones.

**Figure 11:**
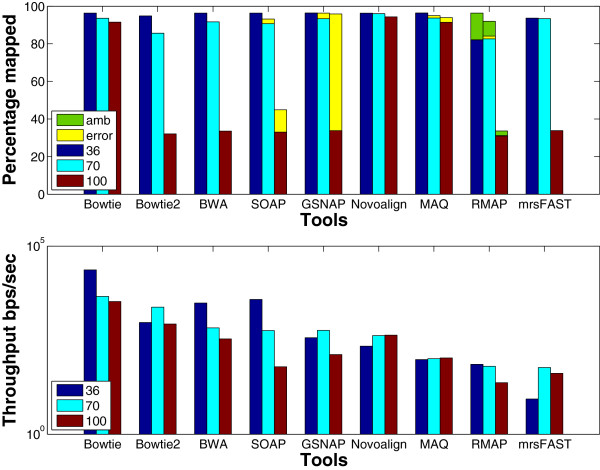
**Effect of changing the read length using a ****ART**** generated data set.** The effect of mapping 1 million reads extracted by ART from the mouse genome version mm9 while changing the read length from 36 to 100.

Additionally, we carried out the same experiment on synthetic data sets generated by the ART tool. We did not carry out the experiment on a real data set due to the lack of proper real data sets that have different read lengths, have exactly the same coverage, generated by the same sequencer, and extracted from the same tissue. The results for this experiment are shown in Figure 11. Similar to wgsim results, the *error* percentage increases with the increase in the read length for GSNAP and SOAP2. Interestingly, the percentage of mapped reads for Bowtie, MAQ, and Novoalign are not significantly affected with the increase in the read length in comparison to the other tools. This is due to the fact that the longer the read is the smaller the quality score becomes for the bases at the end of the reads [49]. Therefore, Bowtie, MAQ, and Novoalign can map the reads with more mismatches while maintaining the same quality threshold.

**Figure 12:**
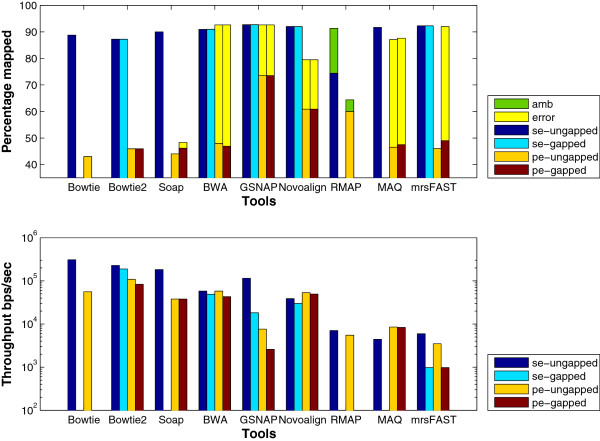
**Effect of using paired-end data using a ****wgsim**** synthetic data set.** The effect of mapping paired-end reads of length 70 to the Human genome. 1 million reads were used to test RMAP and MAQ while 16 million reads were used to test the other tools. SE and PE refer to single end and paired end, respectively. *Error* is only provided for PE due to exceeding the allowed insert size mrsFAST is used for the ungapped alignment and mrFAST is used for the gapped one.

**Paired-end**: Mapping paired-end reads affects the performance of the tools due to the added constraint of mapping both ends within a maximum insert size. Therefore, in this experiment, we want to understand how the performance of the tools is affected while mapping paired-end reads instead of single-end ones. The results in Figure 12 (ungapped bars) show that the throughput decreases for all of the tools while mapping paired-end reads, except for BWA which was able to maintain almost the same throughput while MAQ had a small increase. Even though all of the algorithms work by finding mapping locations for each end alone and then finding the best pair, GSNAP was the only tool to face a drop by 90% in the throughput. Additionally, the percentage of mapped reads is less when mapping paired-end read due to applying the same mapping criteria for single-end reads on paired-end reads.

Even though the maximum insert size was 500, tools such as BWA, SOAP, and GSNAP mapped paired-end reads while exceeding the maximum insert size, except for Novoalign that explicitly requires the user to set the standard deviation for the insert size.

**Figure 13:**
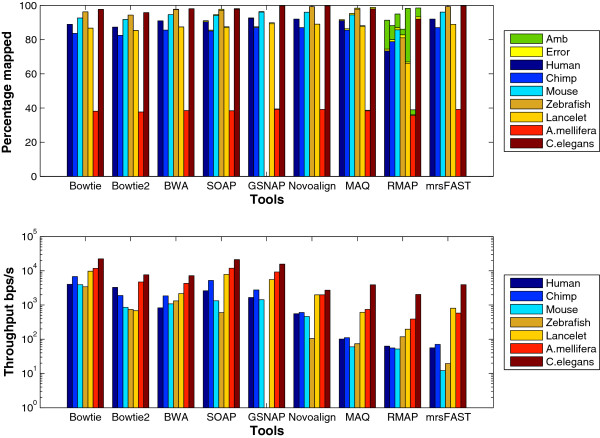
**Effect of changing the genome type using ****wgsim**** generated synthetic data set.** 16 million reads of length 70 bps were generated from the Human, Zebrafish, Lancelet, Chimpanzee, A. mellifera, and C. elegans genomes using wgsim for this test. 1 million reads were used for MAQ and RMAP.

**Genome type**: To capture the effect of the genome type, we designed an experiment in which the Human, Chimpanzee, Mouse, Zebrafish, Lancelet, A. mellifera, and C. elegans genomes were used as reference genomes. The sizes of these genomes are 3.1Gbps, 3.0Gpbs, 2.5Gbps, 1.5Gbps, 0.9Gbps, 0.57Gbps, and 0.1Gbps, respectively. Theoretically, for genome indexing based tools, the throughput is expected to slightly increase with the decrease in the genome size. However, the results in Figure 13 show that some tools do not act as expected. For instance, there is a difference in the throughput between the Chimpanzee and the Human genomes even though their sizes are similar. In addition, SOAP2’s and Novoalign’s throughput decreases significantly for the Zebrafish genome while GSNAP did not finish its run on the same genome albeit running for two days. The reason for this behavior is the large repetition rate in the Zebrafish genome. For instance, while mapping 1 million randomly generated reads from the Zebrafish genome, around 600 reads were mapped to more than 100,000 locations in comparison to the Lancelet with the maximum number of locations is around 10,000 for only 1 read. Additionally, mrsFAST detects more than 8 billions locations when mapping the Zebrafish data set while mapping reads to the Zebrafish genome where it detected only 24 millions when mapping the Lancelet data set. Hence, for GSNAP, the large repetition rates lead to long genomic position lists; resulting in a significant slow down of GSNAP. Another interesting result is the ability of most of the tools to map more than 96% of the Zebrafish data set compared to around 91% of the Human and 89% for the Lancelet data sets. The large mapping percentage is also due to the large repetition rate. Hence, due to synthetically generating the reads, large number of reads are generated from the repeated regions. As a result, the probability of finding a mapping location increases. In addition to the above results, it is also noticed that Bowtie scales better than Bowtie2 on different genomes. Moreover, MAQ’s throughput for the C. elegans genome is larger than Novoalign while maintaining a comparable mapping percentage. Therefore, read indexing based tools might perform better than some genome indexing based tools for small genomes albeit being very slow for large genomes.

**Figure 14:**
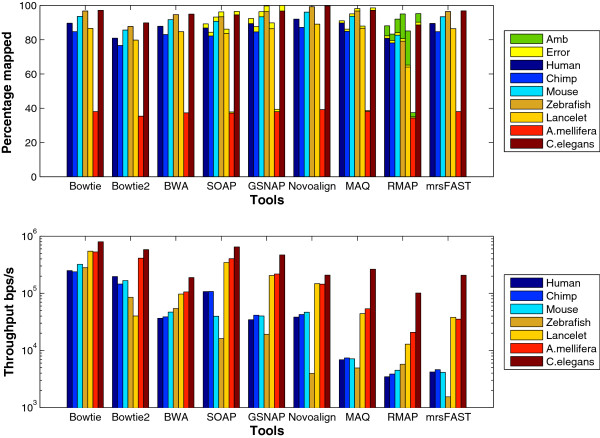
**Effect of changing the genome type using ****ART****generated synthetic data set.** 1 million reads of length 70bps were generated from the Human, Zebrafish, Lancelet, Chimpanzee, A. mellifera, and C. elegans genomes using ART.

To further understand the behavior of the tools, we generated a data set of 1 million reads using ART. Figure 14 shows the results using the ART data sets. Similar to wgsim results, SOAP2 and Novoalign still encounter a significant decrease in the throughput when mapping the Zebrafish data set. Additionally, Bowtie still scales better than Bowtie2 with the different genomes. Interestingly, GSNAP finished its run on the Zebrafish data set even though it still faces a decrease in the throughput. On the other hand, unlike wgsim results, mrsFAST encounters a decrease in the throughput when mapping the Zebrafish data set. It is not obvious why mrsFAST encounters such a decrease even though its performance on the other genomes remains the same regardless of using wgsim or ART.

In general, the throughput of the tools increases when using ART instead of wgsim data sets. However, the relative performance between the tools and the different genomes is still the same.

### Algorithmic features

**Figure 15:**
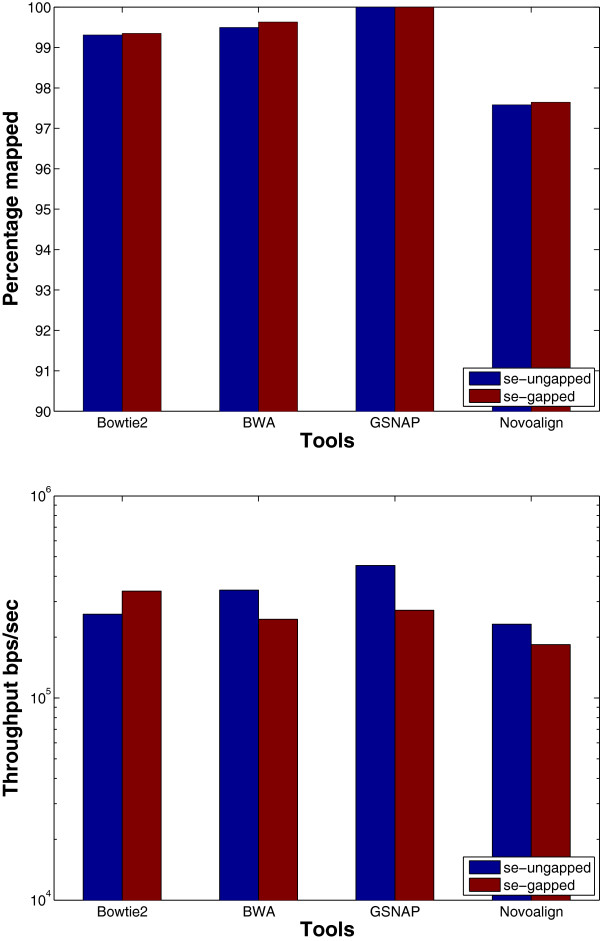
**Effect of enabling gapped alignment using a real data set.** mRNA data set of 1 million reads extracted from the Spretus mouse strain is used in this experiment and mapped on the mouse genome version mm9.

**Gapped alignment** should improve the mapping percentage albeit decreasing the throughput. We designed an experiment to understand the effect of gapped alignment. Tools were used to map synthetically generated reads of length 70 to the Human genome while allowing one gap of length 3. However, mrFAST does not provide any option to limit the gap size. The results in Figure 12 show that the mapping percentage increases by 4% for SOAP2 and 1.5% for mrFAST in case of gapped alignment, while there is no change for BWA and GSNAP. However, there is a drop of 15% and 75% in the throughput for BWA and GSNAP, respectively. The decrease for GSNAP is due to the overhead added to the algorithm to find pairs of candidate regions that co-localize within a maximum allowed gap size. The algorithm tries to find a crossover between the two regions without exceeding the maximum number of mismatches leading to a significant decrease in the throughput. Interestingly, the decrease in the throughput is less for the real data set as shown in Figure 15. However, the decrease is still larger than the decrease in the throughput for the remaining tools.

For the real data set, mrsFAST (mrFAST) is not included in the results since the minimum allowed window size in the indexing phase does not guarantee a full sensitivity for mrFAST.

### Scalability

**Figure 16:**
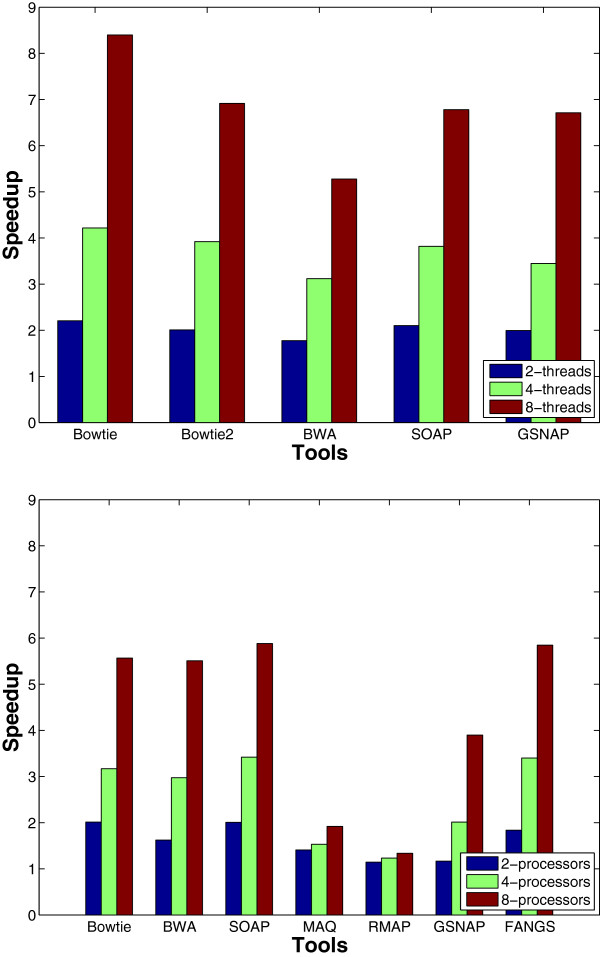
**Speedup when using multithreading and multiprocessing.** 16 million reads of length 125 were mapped to the Human genome while using multithreading (the upper figure) or multiprocessing (the lower figure).

In this experiment, we tested the multithreading behavior. In addition, pMap [50] was used to run multiple instances of each tool on a number of processors on a single node to test the multiprocessing effect. pMap is an open-source MPI-based tool that enables parallelization of existing short sequence mapping tools by partitioning the reads and distributing the work among the different processors. A single node was used in the multiprocessing experiment to understand the effect of a good implementation of multithreading. The results for both experiments are given in Figure 16. We can observe from the multithreading results that the tools had almost a linear speedup up to 4 threads. However, when increasing to 8 threads, Bowtie was the only tool to achieve 8x speedup. In addition, BWA had a similar speedup in both multithreading and multiprocessing. For the multiprocessing experiment, FANGS achieved almost a 6x speedup while there was a small improvement for MAQ and RMAP. For the remaining tools, most of them were able to maintain more than a 5x speedup for 8 processors, however this is less than a linear speedup. One reason for this degradation is the overhead of the distribution and merging steps required by distributed memory systems. As expected, we can notice that multithreading provides almost a linear speedup, however, it is limited by the number of cores.

In general, using multiprocessing provides more degrees of freedom by parallelizing tools that do not support multithreading and by making use of the available computational resources.

Another important observation is the effect of the indexing method on the total speedup. Read indexing based tools did not have any significant speedup in comparison to the genome indexing based ones which had more than 5x speedup. Therefore, genome indexing is more efficient in case of designing a read partitioning parallelism based tool.

### Accuracy evaluation

The aim of this experiment is to evaluate the percentage of reads each tool actually maps out of the set of the mappable reads. A read is mappable if the distance between the read and its original genomic location does not violate the mapping criteria. In this experiment, the reads were generated using ART to measure the sensitivity of the tools in case of varying the distribution of mismatches. The used mapping criterion was fixed to five mismatches for Bowtie2, SOAP, GSNAP, BWA, mrsFAST, and RMAP. For the remaining tools, a quality threshold of 100 was used. In general, gapped alignment was disabled. The results given in Table 2 show that Bowtie did not map around 0.14% of the set of the mappable reads (i.e., *false negatives*) while Bowtie2 did not map around 7.71%. Moreover, Bowtie mapped 93% of the reads while Bowtie2 only mapped 85%. Nevertheless, the sensitivity of both tools can be increased by changing the default options at the expense of significantly decreasing the throughput. Interestingly, BWA, SOAP2, and mrsFAST mapped all of the mappable reads without any *error*.

**Table 2:** Sensitivity evaluation of the different tools

		**Bowtie**	**Bowtie2**	**BWA**	**SOAP2**	**MAQ**	**RMAP**	**GSNAP**	**Novoalign**	**mrsFAST**
Mapped	Expected	93.57	93.25	91.29	91.29	93.57	90.12	93.25	96.18	93.25
	Actual	93.43	85.54	91.29	91.29	92.92	82.53	93.25	96.02	93.25
	Error				0.73			0.03		
Unmapped	Expected	6.43	6.75	8.71	8.71	6.43	9.88	6.75	3.82	6.75
	Actual	6.25	6.68	8.32	6.83	5.08	8.29	3.66	3.81	6.62
	Error				1.73	1.25	1.5	2.97		
Reported mapped		93.61	85.61	91.68	93.17	94.27	84.11	96.34	96.03	93.38
Reported correct		93.61	85.61	91.68	90.71	93.02	82.61	93.37	96.03	93.38

Evaluating the sensitivity of the tools on a data set of 1 million reads of length 70 generated by ART. The numbers are in percentage. The Reported mapped percentage is the total percentage of reads mapped by each tool. It is equal to Actual Mapped + (Expected Unmapped- Actual Unmapped) while Reported correct is the total number of correctly mapped reads.

In general, the tools were able to map a percentage of the unmappable reads, however, it was mapped with a large *error* percentage. For instance, even though GSNAP mapped around 3% of the unmappable reads, only 0.03% of them were correctly mapped. Therefore, even though GSNAP maps the largest percentage of reads, other tools such as BWA and Novoalign are more accurate and precise than GSNAP.

It is important to note that the percentage of reads mapped from the unmappable reads is similar to the percentage of *incorrectly mapped reads-relaxed* given in Ruffalo et al. work [32]. However, they define a read to be unmappable if it has a mapping quality less than a certain threshold while we consider it as unmappable if it violates the mapping criteria for the tool.

### Rabema evaluation

The aim of this experiment is to evaluate the tools based on the number of reads with a specified error rate the tool has been able to map. Unlike the previous experiment, this experiment does not take into consideration how each tool works. Therefore, it is similar to evaluating the efficiency of the different mapping techniques (i.e., seeding vs. non-seeding, quality scores vs. mismatches). The experiment is performed on a synthetic data set of length 100 extracted from the Human genome using ART. The maximum allowed error rate was 5%, i.e., 5 mismatches in that case. The results for this experiment are shown in Table 3. Rabema takes the output SAM file from each tool as the input. However, MAQ and RMAP do not create the output in the SAM format. Therefore, there are not included. Additionally, GSNAP results are not included since the output GSNAP SAM file contains messed up quality scores.

**Table 3:** Rabema evaluation

**Error**	**#Reads**	**Bowtie**	**Bowtie2**	**BWA**	**SOAP2**	**Novoalign**	**mrsFAST**
0	832	100	100	100	100	97.24	100
1	6316	96.99	100	100	100	98.29	100
2	23495	97.30	97.16	100	99.97	98.70	100
3	55941	97.00	95.92	99.85	95.78	98.84	100
4	98063	96.48	94.22	99.49	96.43	99.02	100
5	135096	95.63	91.14	98.76	97.34	99.12	100

Rabema evaluation results on the different tools using a data set of 1 million reads of length 100 extracted from the Human genome using ART. The maximum allowed error is 5% (i.e., 5 mismatches in this case). #Reads is the number of reads expected to be mapped with certain Error. The remaining columns for the tools show the percentage of reads detected by each tool out of the #Reads. Invalid mappings (i.e., reads mapped with errors more than the assigned error rate threshold) for Bowtie and Novoalign are 567,531 and 587,542 reads, respectively.

As shown in the results, both Novoalign and Bowtie are evaluated as mapping invalid reads. This is because Rabema does not take the quality scores into consideration and just calculate the edit distance. Therefore, from the mismatches perspective, the reads have more than 5 mismatches. However, from the quality threshold perspective, they have a quality threshold less than the specified one. Therefore, at the end, they are valid mappings.

In general, BWA has been able to detect almost all of the reads with the correct error rate. This suggest that most of the mismatches exist at the end of the read. In addition, the seeding technique is a valid method specially if it can speed up the mapping process.On the other hand, even though SOAP2 is a seed based tool, similar to BWA, it could not detect as much correct reads as BWA. Bowtie2 missed some of the reads, however, it can detect them by changing its sensitivity at the cost of increasing the running time. On the other hand, mrsFAST mapped all of the reads with the correct error rate since it is a full sensitive mapper.

### Use case: SNP calling

The aim of this experiment is to understand how the different mapping techniques affect the quality of SNP calling. The tools were used to map an mRNA dataset of 23 million reads extracted from the Spretus mouse strain. Then Partek [51], a genomic suite developed to analyze NGS data, is used to detect SNPs. The mouse genome version mm9 was used as the reference genome in this experiment. A quality threshold of 70 was used for Bowtie and Novoalign while the remaining tools were allowed 5 mismatches. In addition, gapped alignment was enabled for Bowtie2, BWA, GSNAP, and Novoalign. Table 4 shows the results for this experiment. The SNP detection step was done for GSNAP and SOAP2 after filtering out the erroneous reads. The log-odd ratio represents how accurate the SNP is. The small log-odds ratio for some of the SNPs is due to either the small number of reads that supports that SNP or the mixed genotype calls. We can observe that there is a large number of accurately detected SNPs. This is expected due to the high divergence of the Spretus strain from other mice strains. For the sake of completeness, we are including the whole number of detected SNPs, however, in our analysis, we focus only on the number of accurately detected SNPs shown in the last column. The results show that GSNAP detected the largest number of accurate SNPs while Novoalign detected the smallest. In addition, more than 94% of the highly accurate SNPs detected by Novoalign were also detected by the other tools (not shown). To further understand the reason for the low number of SNPs detected by Bowtie and Novoalign, we carried out the same experiment while using a quality threshold of 100. The number of highly accurate SNPs increased to 1474 and 1100 for Bowtie and Novoalign, respectively. Moreover, the reads with more than 5 mismatches did not contribute to the increase in the number of SNPs. This is due to the fact that SNPs have a high base quality score. Therefore, a read with a SNP would be sequenced with a small number of errors.

**Table 4:** SNP calling results

**Tools**	**Log-odds ratio**											
	**5**	**100**	**200**	**300**	**400**	**500**	**600**	**700**	**800**	**900**	**1000**	**1000000**
Bowtie	89479	24337	5082	2231	1076	648	426	281	0	0	0	1171
Bowtie2	200914	62178	10018	4200	2052	1156	767	537	0	0	0	2035
BWA	192050	52115	9028	4049	1894	1087	737	525	0	0	0	2067
SOAP2	174475	49302	8552	3824	1837	1030	704	508	0	0	0	1941
Novoalign	69798	17586	4061	1875	936	519	363	252	0	0	0	941
GSNAP	207920	69015	11416	4928	2482	1325	971	617	0	0	0	2602

SNP calling results when using the different tools. Each row represents a different tool while each column shows the number of SNPs detected with a log-odds ratio, which is a measure of the accuracy of the detected SNP, centered around the given values. The larger the log-odds ratio is, the more accurate the detected SNP becomes.

## Conclusion

There have been many studies carried out to analyze the performance of short sequence mapping tools and choose the best tool among them. However, the analysis of short sequence mapping tools is still an active problem with many aspects have not been addressed yet. In this work, we provided a benchmarking study for short sequence mapping tools while tackling different aspects that have not been covered by previous studies. We mainly focused on studying the effect of different input properties, algorithmic features, and changing the default options on the performance of the different tools. Additionally, we provided a set of benchmarking tests which extensively analyze the performance of the different tools. Each of the benchmarking tests stresses on a different aspect. The benchmarking tests were further applied on a variety of short sequence mapping tools, namely, Bowtie, Bowtie2, BWA, SOAP2, MAQ, RMAP, GSNAP, Novoalign, mrsFAST (mrFAST), and FANGS.

The experiments show that some tools report an *error* percentage (i.e., reads mapped while violating the mapping criteria). Among these tools are GSNAP and SOAP2. GSNAP reported the highest *error* percentage in the experiments. Additionally, the *error* increases with the read length and it decreases with the the number of mismatches. Nevertheless, GSNAP was one of the tools which reports the largest mapping percentage in most of the experiments even after filtering out the *error* reads. The main reason for mapping more reads is allowing any number of mismatches in the seed part. From a real application perspective, GSNAP’s filtered output helped in detecting the largest number of SNPs.

The evaluation of Bowtie, Bowtie2, BWA, mrsFAST, and Novoalign show their ability to correctly map the reads. Moreover, Novoalign mapped the largest percentage of reads, similar to GSNAP, specially for highly repeated genomes. However, it maintained the lowest throughput among the genome indexing tools in most of the experiments.

mrsFAST’s throughput is highly affected by the read length and the number of mismatches. Our experiments show that it is better to use mrsFAST for longer reads. It can also be used for short reads but only with a small number of mismatches.

In general, genome indexing based tools performed better than read indexing tools in all of the experiments. However, MAQ was faster than Novoalign for small genomes. Therefore there is a potential for read indexing tools to be used for small genomes. In addition to providing the worst performance, read indexing does not provide any significant speedup in case of using read partitioning based parallelism. Therefore, the read indexing method is not preferred when designing a new read partitioning mapping tools.

Interestingly, the genome type experiment revealed many strengths and weaknesses for the tools. For instance, the performance of SOAP2, GSNAP, and Novoalign is highly dependent on the genome type; the throughput decreased significantly for the Zebrafish genome. This is due to the large repetition rate on the Zebrafish genome. In addition, the tools behaved differently on the Human and the Chimpanzee genomes albeit having comparable genome sizes. The results of the genome type experiment suggest that the different properties of the genomes affect the performance of the tools. Therefore, further investigations are required to understand the different properties of the genomes and their effect on the different mapping techniques.

Even though there are differences between the results for the real data sets and the synthetic ones, both experiments are important as they give us a different perspective when comparing between the tools. The control on the number of mismatches for the wgsim synthetic data allows us to know exactly what the throughput of each tool is while looking for exactly the same number of mismatches. Therefore, it becomes easier to understand why a tool is faster than another one or why a tool seems to map more reads than the other ones. At the same time, it is important to look at the behavior of the tools in case of real data and real-like synthetic data (e.g., ART) to further understand how they behave in the real world. For instance, for the number of mismatches experiment, even though Bowtie looks like it maps a percentage of reads similar to the other tools in case of 7 t-mms, it actually maps the reads with a maximum of 4 t-mms. Therefore, the output mappings are more accurate than the other tools.

In general, there is no *the-best* tool among all of the tools; each tool was *the-best* in certain conditions. The short sequence mapping problem is still an active problem and new tools are needed to be developed. However, these new tools should be application-specific. By taking the target application into consideration, more accurate results can be obtained. For instance, for genome assembly, we can analyze the reference genome and estimate the number of reads that can be mapped for the different regions (e.g., repeated regions) based on the coverage information in the sequencing process. Another example for an application with very specific properties is the mapping of RNA-Seq data which contain short sequences for the exon regions rather than intron regions for the genome. Therefore, for well-studied genomes, if a small number of reads where mapped to different intron regions, we can expect them to be wrongly mapped and look for other mapping locations with more number of mismatches or less mapping quality.

## Availability

Links to tools, datasets, parameters used in each individual tool and test, and the code used to verify the tools are available at http://bmi.osu.edu/hpc/software/benchmark/.

## Competing interests

The authors declare that there are no competing interests.

## Authors’ contributions

AH and DB designed the experiments, AH carried out experiments, wrote the first draft of the manuscript. AET provided and analyzed SNP dataset. UVC led the project, helped with the design and interpretation of the experiments, reviewed and revised the manuscript. All authors read and approved the final manuscript.
